# Pulmonary inflammatory Myofibroblastic tumor indistinguishable from tuberculosis: a case report in a five-year-old child with hemoptysis

**DOI:** 10.1186/s13019-017-0670-9

**Published:** 2017-12-08

**Authors:** Tao Zuo, Jun Fu, Zhengyi Ni, Baojun Chen

**Affiliations:** 1 Department of Thoracic Surgery, The Central Hospital of Wuhan, Tongji Medical College, Huazhong University of Science and Technology, Wuhan, 430022 Hubei Province People’s Republic of China; 2 Department of Thoracic Surgery, Wuhan Medical Treatment Center, 1 Yintan Road, Dongxihu District, Wuhan, 430023 Hubei Province People’s Republic of China

**Keywords:** Pulmonary inflammatory myofibroblastic tumor, Tuberculosis

## Abstract

**Background:**

Pulmonary inflammatory myofibroblastic tumor (PIMT) is a rare disease in China and its incidence is much lower than that of tuberculosis. PIMT accounts for only 0.04–1.2% of all lung tumors. PIMT can occurs in any age and nearly every part of the body. The clinical symptoms and radiological features of PIMT are nonspecific. Diagnosis is only made on the basis of histopathologic or immunohistochemical evaluation of the postoperation resected tissue. The therapeutic approach to PIMT should rely mainly on complete surgical resection.

**Case presentation:**

We report a case of PIMT with hemoptysis. The girl was misdiagnosed with tuberculosis and treated with anti-tuberculous drugs for a long period of time. A right upper and middle lobectomy was performed and further assessment of the tissue demonstrated a pathologic diagnosis of PIMT.

**Conclusions:**

Despite a high incidence of tuberculosis, we must consider the possibility of PIMTs in such cases to prevent misdiagnosis and mistreatment.

## Background

Inflammatory myofibroblastic tumor (IMT) is an uncommon pathological entity with variable locations, such as spleen, breast, maxillary sinus, epididymis, xcentral nervous system, and soft tissues [1, 2].The highest incidence occurs in the lungs of children and adolescents. Pulmonary inflammatory myofibroblastic tumor (PIMT) accounts for only 0.04–1.2% of all lung tumors [3, 4]. First defined by Brunn in 1939, histologically, it is composed of fascicles of spindle cells with a prominent inflammatory infiltrate [5]. And PIMT is considered as real neoplasm now, because of the proliferation of myofibroblastic cells and the malignant behavior with a high potential for recurrence [6, 7].

Clinical symptoms and radiological features of PIMT are nonspecific. So it is difficult to make the diagnosis before surgical resection. What’s more, it remains a challenge to differentiate PIMT from malignant tuberculosis (TB) and lung tumors. Especially to children, it stands a good chance of being misdiagnosed as tuberculosis in high burden countries (HBCs). Millions of children in HBCs are still dying from tuberculosis, despite being full vaccinated. In China, there are few PIMT patients but many TB patients whose laboratory tests are all or almost negative. And when it is highly suggestive of tuberculosis in clinically presentation but without other evidence, our physicians sometimes use anti-tuberculous treatment (ATT). Herein, we report a case of a girl presented with hemoptysis, misdiagnosed with tuberculosis and treated with antituberculous drugs nearly a year.

## Case presentation

**Fig. 1:**
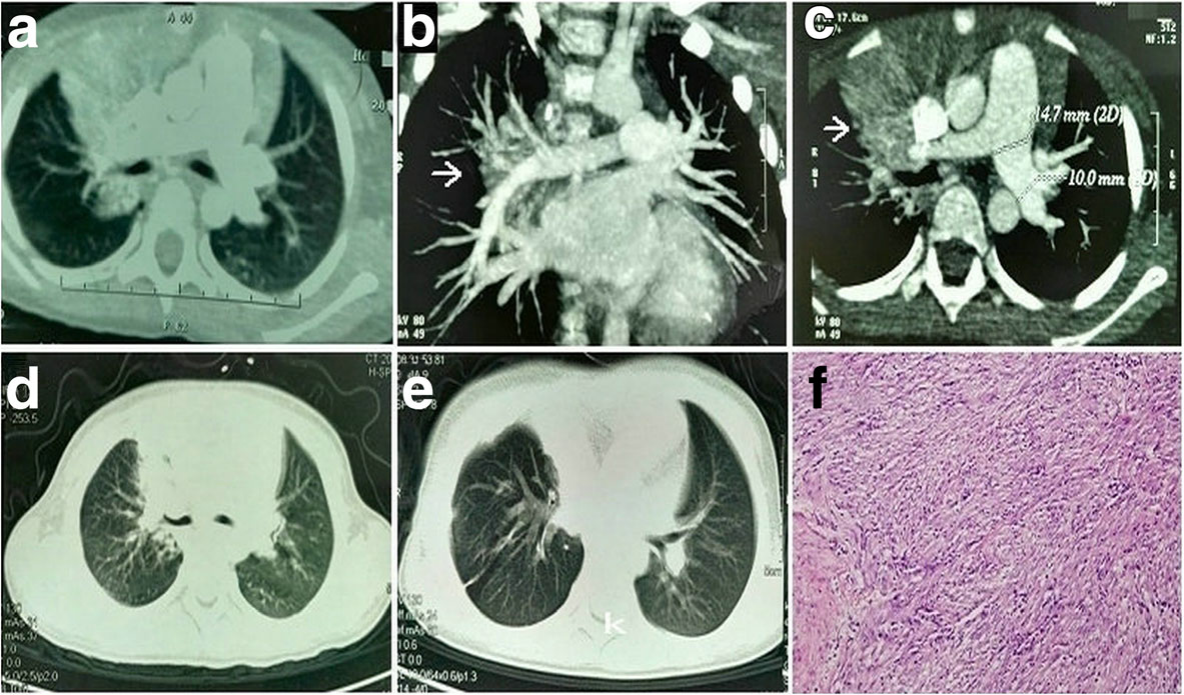
Chest computed tomography show right upper and middle lobes pneumonia and right middle lobe atelectasis (**a**). And a pulmonary hilar mass involve the right pulmonary artery (**b**, **c**). The review of thoracic CT scans revealed the mass did not progress during the period of ATT (**d**). A postoperative thoracic CT showed there is no recurrence after six months. Photomicrograph of the tumor specimen indicated spindle cells with infiltration of lymphocytes, plasma cells, and foamy histiocytes (**f**)

A 5-year-old girl presented to our institution with complaint of twice hemoptysis over the past year. She suffered the several bloody sputa at the first time a year ago, and then she visited a children’s hospital at once. Thoracic computed tomography (CT) showed right upper and middle lobes pneumonia and right middle lobe atelectasis (Fig. 1a–c). Bronchoscopic examination demonstrated mucosal hyperemia on the right side of bronchus, hilar swell and stenosis in the right upper and middle lobes with bleeding when palpated. Brush biopsy in bronchoscopy and pathogenic bacteria culture in bronchoalveolar lavage fluid (BALF) all were negative. The serum Mycobacterium tuberculosis (MTB)-specific human antibodies and antigens 38KD, LAM were positive. Then she visited the tuberculosis physicians. They performed Mycobacterium tuberculosis polymerase chain reaction (PCR), tuberculin skin testing (TST) of Bacille Calmette-Guerin (BCG) and T-spot.TB, all of which revealed negative results. According to the past contact history (her grandfather suffered from TB) and laboratory result, physicians diagnosed this girl as suspected pulmonary and bronchial tuberculosis. She was taken anti-tuberculousis treatment (ATT, isoniazid, rifampicin, ethambutol and pyrazinamide,) for 11 months before this presentation. The reviews of thoracic CT scans examinations showed the disease was stable during the period of ATT (Fig. 1d). She presented to our institution when suffered the second hemoptysis. About 100–200 ml of arterial blood was intermittent coughed up in an hour. The physical examination showed no abnormality and routine hematological and biochemical parameters were roughly within normal range. After confirming that hemostatics treatment by drugs is invalid, the patient underwent exploratory thoracotomy at first. General anesthesia and double cavity intubation was used during operation, then bronchoscopic examination indicated active bleeding in the right middle lobe. A right upper and middle lobectomy was performed, since a mass locate in the right upper and middle lobe and the middle lobe were atelectasis. It was reported as PIMT by histologic examination (Fig. 1f). The patient has no current complaint, and a postoperative thoracic CT showed there is no emerging lesion after six months (Fig. 1e).

## Discussion

IMT is defined by the World Health Organization (WHO) in 1994 as a soft tissue tumor composed of fibrous tissues, myofibroblasts and inflammatory infiltration, predominantly histiocytes and plasma cells. And it was named as plasma cell granuloma, xanthogranuloma, inflammatory pseudotumor, and fibrous histiocytoma before [8]. Though arising in various anatomic locations, IMT most frequently occurs in the lungs. The cause of PIMT is still unclear. It was likely to have been associated with recurrent infections [9, 10]. Approximately 50% of all IMTs showed a chromosomal translocation of the anaplastic lymphoma kinase (ALK) gene (chromosome 2p23) with a partner gene (nucleophosmin or others) locus with potentially aberrant kinase expression [11, 12]. Hornick et al. demonstrated that expression of ROS1 correlated with ROS1 gene rearrangement in IMT [13].

PIMT can occurs in any age, but usually be seen in children and adolescent. There was barely difference in the proportion of males and females who sufferred from PIMT.

The common presenting complaints with PIMT are nonspecific symptoms, such as cough, expectoration, fever, shortness of breath, chest pain, hemoptysis, and fatigue. PIMT is divided into two types: One is invasive and another one is noninvasive. Invasive IMT usually occurs in younger patients and may reach larger size invading surrounding structures. Noninvasive PIMT presented as ill-defined or irregular lesions [14]. In addition, others, including calcification, cavity, necrosis, obstructive atelectasis and pneumonia can also been found.

The imaging characteristics of PIMT are variable and nonspecific, such as calcification, cavity, necrosis, obstructive atelectasis and pneumonia. And it is similar to lung cancer, tuberculosis, inflammatory pseudotumors and so on. The most typical image is a solitary, well-defined, in the periphery of the lung on CT graph. And Wu et al. revealed that PIMT increased in attenuation by between 12 and 79.1 Hounsfield units (HU; mean, 38.5 ± 22.8 HU) in the arterial phase, and between 30.4 and 57.9 HU (mean, 44.2 ± 10.2 HU) in the venous phase [15]. Some patients can be found calcification in the centre or center-biased of lesins.

Histopathologically, IMTs can be divided into three histologic types: organizing pneumonia pattern, fibrous histiocytic pattern, and lymphohistiocytic pattern. As the most common type, the second one is characterized by spindle shaped myofibroblasts arranged in whorls, including myofibroblasts and fibroblasts, arranged in a fascicular or storiform manner, and surrounded by chronic inflammatory cell infiltration [16]. On immunohistochemistry tumor cells exhibit strong diffuse positivity with smooth muscle actin and vimentin, and are negative for cytokeratin, CD34 and S100 [17].

It remains a difficulty to diagnose PIMT. Because it is easily misdiagnosed as inflammatory lesions or tumors of mesenchymal origins. The definitive diagnosis always can not be made by perioperative CT-guided percutaneous lung biopsy and intraoperative rapid frozen section biopsies. It is based on histopathologic or immunohistochemical evaluation of the preoperative resected tissue.

It is not rare to misdiagnosed PIMT as tuberculosis in HBCs. A large numbe of latent mycobacterium tuberculosis infections lead to positive tuberculosis blood test, such as Mycobacterium tuberculosis (MTB)-specific human antibodies and antigens, TST, T-spot, etc. There is only slight agreement and high variability among all diagnostic instruments. And the symptoms of tuberculosis in children are atypical. So it is easy to delay the diagnosis and affect the treatment. In this case, the girl was regular treatment with antituberculousis drugs for 11 months in the local Centre for Disease Prevention and Control. The positive tuberculosis blood test and improvement of her condition during the period of ATT affect diagnosis.

The therapeutic approach to PIMT should rely mainly on complete surgical resection. It was reported that 6.6–13% of PIMT following the resection may locally recur [11].

The incomplete resections should probably lead to recurrence. The reappearance of clinical manifestation always foreshow the recurrence. Corticosteroids can be used in children with unresectable disease. Chemotherapy and radiotherapy may be active for multifocal, invasive lesions and in cases of local recurrence. After surgery, patients should be followed up at short intervals by bronchoscopy and thoracic CT to monitor recurrence [18–20].

## Conclusion

We report a case of hemoptysis as the primary manifestation of PIMT, which is very rare. She was misdiagnosed with tuberculosis and treated with antituberculous medication for a long period of time. Despite a high incidence of tuberculosis, we must consider the possibility of PIMT in such cases to prevent misdiagnosis and mistreatment.

## Abbreviations

ALK: Anaplastic lymphoma kinase
ATT: Anti-tuberculous treatment
BALF: Bronchoalveolar lavage fluid
BCG: Bacille calmette-guerin
CT: Computed tomography
HBCs: High burden countries
HU: Hounsfield units
IMT: Inflammatory myofibroblastic tumor
MTB: Mycobacterium yuberculosis
PCR: Polymerase chain reaction
PIMT: Pulmonary inflammatory myofibroblastic tumor
TB: Tuberculosis
TST: Tuberculin Skin Testing
WHO: World Health Organization
